# Personalized Radiation Therapy in Cancer Pain Management

**DOI:** 10.3390/cancers11030390

**Published:** 2019-03-19

**Authors:** Ewa Sierko, Dominika Hempel, Konrad Zuzda, Marek Z. Wojtukiewicz

**Affiliations:** 1 Department of Oncology, Medical University of Bialystok, 15-027 Białystok, Poland; domhem@wp.pl (D.H.); mzwojtukiewicz@gmail.com (M.Z.W.); 2 Department of Radiation Therapy, Comprehensive Cancer Center of Białystok, 15-027 Bialystok, Poland; 3 Student Scientific Association Affiliated with Department of Oncology, Medical University of Bialystok, 15-027 Bialystok, Poland; konrad.zuzda@outlook.com

**Keywords:** cancer pain, pain management, radiation therapy

## Abstract

The majority of advanced cancer patients suffer from pain, which severely deteriorates their quality of life. Apart from analgesics, bisphosphonates, and invasive methods of analgesic treatment (e.g., intraspinal and epidural analgesics or neurolytic blockades), radiation therapy plays an important role in pain alleviation. It is delivered to a growing primary tumour, lymph nodes, or distant metastatic sites, producing pain of various intensity. Currently, different regiments of radiation therapy methods and techniques and various radiation dose fractionations are incorporated into the clinical practice. These include palliative radiation therapy, conventional external beam radiation therapy, as well as modern techniques of intensity modulated radiation therapy, volumetrically modulated arch therapy, stereotactic radiosurgery or stereotactic body radiation therapy, and brachytherapy or radionuclide treatment (e.g., radium-223, strontium-89 for multiple painful osseous metastases). The review describes the possibilities and effectiveness of individual patient-tailored conventional and innovative radiation therapy approaches aiming at pain relief in cancer patients.

## 1. Introduction

The majority of cancer patients suffer from pain during course of their disease [1]. Pain may be a result of a growing primary tumour, metastases in lymph nodes, but predominately it occurs in patients experiencing distant metastases, particularly to the skeletal system. Pain management can significantly improve patients’ quality of life [2]. Antineoplastic treatment, e.g., radiation therapy, may also cause transient pain, which has to be managed pharmacologically [3]. Radiation therapy (RT) per se, however, significantly contributes to pain alleviation in cancer patients in multiple clinical scenarios [4,5]. Pain relief after radiation therapy may be achieved in as many as 60–80% of patients [2]. Recently, a strong need of substantial engagement of radiation oncologists in pain relief (among other symptoms produced by advanced disease) in cancer patients was a subject of the American Society for Radiation Oncology (ASTRO) members’ statement [6].

## 2. Painful Bone Metastases

About 50% of all cancer patients will develop bone metastases [7]. The frequency of bone metastases’ occurrence depends on tumour type, with breast, prostate, and lung cancers accounting for 85% of the cases [8,9]. Other primaries which frequently produce bone metastases include urinary bladder, renal, endometrial, and thyroid cancer as well as melanoma [9]. Bone pain may be caused by a local invasion of a metastatic tumour, which leads to remodelling of the microenvironment (changing the equilibrium between the activity of osteoblasts and osteoclast and bone structural degradation), direct nerve root invasion, or an increased release of inflammatory mediators, which stimulate nerve fibres [10,11,12]. Furthermore, a spasm of surrounding muscles may cause discomfort to the patient. Radiotherapy is the most effective treatment for bone metastases [13].

### 2.1. Pathomechanism of Analgesic Effect of Radiation

The exact mechanism of radiation-induced pain relief is unknown. The analgesic effect of radiation is achieved by stimulating ossification, diminishing osteoclasts’ activity in bone microenvironment, and killing cancer cells along with reduced osteolysis, which results in decreasing tumour burden [12,14]. Rapid pain relief (even after 24 h), observed in some patients, indicates a decrease of inflammatory cell activity as well as chemical pain mediators concentration in the radiation field, which play a role in the analgesic effect of radiation [15]. The RT fractionation scheme in patients with bone metastasis influences the level of mineralization, bone density, and recalcification of the irradiated site [16,17], which is associated with pain response. The recalcification rate was lower in the single fraction group (120%) compared to patients who received fractionated RT (173%, *p* < 0.0001) with a slight trend favouring 10 × 3 Gy RT scheme [17]. An association was observed between radiotherapy-driven bone pain relief and low osteoclast activity markers’ (pyridinoline—PYD, and deoxypyridinoline—DPD) concentrations in the urine before and after RT [18]. Re-irradiation for painful bone metastases also influences osteoclast activity visualized by urinary markers—PYD and DPD [19]. Significant differences at the baseline markers’ urinary levels were detected between 40 responders and 69 non-responders to re-irradiation (*p* = 0.03 for PYD and *p* = 0.04 for DPD) [19].

### 2.2. External Beam Radiation Therapy for Painful Bone Metastases

**Figure 1:**
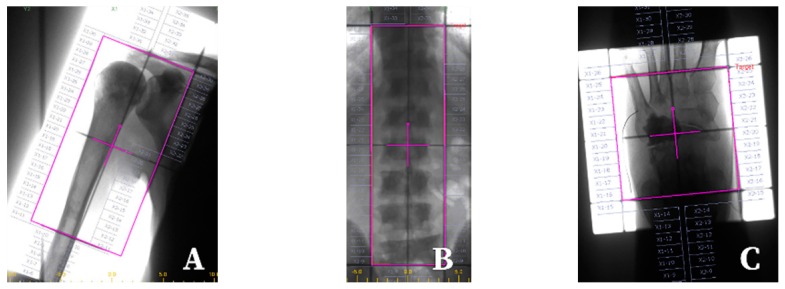
Simulation fields (pink rectangle) for irradiation of painful bone metastases with megavoltage photon external beams: (**A**) humeral bone, (**B**) spine, (**C**) foot bones.

**Figure 2:**
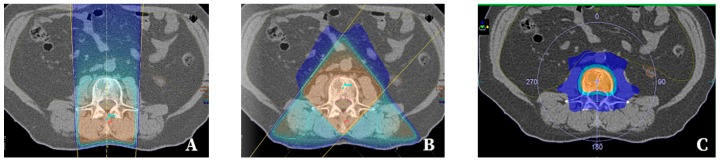
Isodose distribution in three-dimensional conformal radiation therapy (3D-CRT) for painful metastasis in dorsal vertebra: (**A**) one beam, (**B**) two oblique beams, (**C**) volumetrically modulated arch therapy (VMAT).

Radiation therapy with external beams may be delivered using different radiation techniques, the most common of which, for the purpose of irradiation of painful bone metastases, are the following: Two-dimensional radiation therapy (2D-RT) (Figure 1), still used especially in patients with expected short survival, and three-dimensional conformal radiation therapy (3D-CRT) (Figure 2). Modern modifications of the 3D-CRT radiation techniques include intensity modulated radiation therapy (IMRT), volumetrically modulated arch therapy (VMAT), or tomotherapy [20], which allow for better tumour coverage along with sparing surrounding normal tissue, thus decreasing potential toxicity of the treatment (Figure 2). Nowadays, sophisticated stereotactic radiosurgery (SRS) or stereotactic body radiation therapy (SBRT), allowing for delivery of a very high biological dose of radiation for very precisely delineated tumour masses, is increasingly used in selected groups of patients (Figure 2). Qualification of a particular patient for certain techniques and fractionation schedules in clinical practice depends on multiple factors (Table 1).

#### 2.2.1. Conformal Radiation Therapy

The optimal choice of fractionation schedule for the treatment of painful bone metastases is still an unresolved issue. Most skeletal metastases are multiple in nature and only 10% are diagnosed as solitary ones [21].

During the last three decades, many randomized and observational trials focused on the optimal choice of the dose and fractionation regimen for pain relief [9,17,22,23,24,25,26,27,28]. They demonstrated an equivalent analgesic effect and durability of a single 8 Gray (Gy) fraction with multiple fractionation schedules, such as 30 Gy delivered in 10 fractions, 24 Gy given in 6 fractions, or 20 Gy in 5 fractions (Table 2) [9,17,21,22,23,24,25,26,27,28].

A meta-analysis of 25 randomized clinical trials revealed that up to 80% of patients with uncomplicated bone metastases experience analgesic response to external beam radiotherapy and 25–30% of patients achieve complete pain relief within 3–4 weeks after radiation therapy [2]. Uncomplicated bone metastases include metastatic tumour masses without massive infiltration towards soft tissue, characterized by a low risk of imminent pathological fracture and no evidence of spinal cord compression or cauda equina compression, and which were not previously irradiated [15]. Pain control persisting 3–6 months after radiation therapy, however, is experienced by 35% of patients only [2]. Of note, the re-treatment rate to in-field pain recurrence was higher in those patients who received single-fraction regimens than in those treated with multiple fraction schemes (20% vs. 8%, *p* < 0.00001) [2]. Yet, it is unclear if this was due to lower durability of pain control or to the physician’s comfort with re-treating after a lower initial radiation dose. It should be emphasized that single fraction treatment was associated with lower acute toxicity (nausea and vomiting, diarrhoea, fatigue, or skin toxicity). On the other hand, single fraction treatment was related to a higher rate of spinal cord compression and pathological fractures, but the difference did not reach statistical significance. The limitation of this meta-analysis are nonuniform primary end-points in different studies [2].

Despite the same efficacy of a single 8 Gy fraction, compared with multiple-fraction regimens and available published guidelines [11,15], to date this single fraction schedule is underused in the clinical practice to treat painful bone metastases [33]. Fischer-Valuck et al. [34], however, report increased usage of short course radiation therapy (1 × 8 Gy or 5 × 4 Gy) over long course treatment in 2014, compared to 2000, particularly in academic centres, in elderly prostate cancer patients living more than 15 miles from the treatment facility. In fact, single radiation fraction treatment allows patients to receive the planning procedure and radiation therapy within the same day, which is of particular importance for those with poor performance status [35,36].

The decision making should be based on treatment-related costs as well. In this context, some study results are of interest. A Dutch randomized controlled trial enrolling 1157 patients revealed that the cost of radiation therapy, including re-treatments and other nonmedical costs, was significantly lower for a single fraction regimen compared to a multiple fraction schedule ($2438 vs. $3311, *p* < 0.001) [37]. Similar financial results were obtained by the Radiation Therapy Oncology Group (RTOG, trial 9714) [38]. The New Zealand study revealed that single fraction radiotherapy costs per patient amounted to NZ$1344 and were lower than the costs of multiple fractionation regimens for prostate cancer patients [39]. Furthermore, SEER (The Surveillance, Epidemiology and End Results) analysis found a difference of $3094 between single fraction and multiple fractionations (10 fractions or more) [40]. It has been estimated that an absolute increase of 10% of single fraction radiation therapy administration for painful bone metastases in prostate cancer patients’ population could generate more than $70 million per year in health cost savings [41,42,43]. Of interest, in India the average distance travelled per day by a patient for radiation treatment is about 100 km [27], which means that patients undergoing single fraction treatment will travel 100 km for a complete radiation regimen, whereas patients undergoing 10-fraction radiation therapy will travel approx. 1000 km. It increases the costs from Rs1010 to Rs9700 per patient [27,37,38,39,40,41,42,43].

The neuropathic pain component represents a special issue. It occurs in 19–39% of cancer patients [44,45,46]. Pain management with pharmacotherapy may be troublesome, although it is the mainstay of the treatment [46,47]. Of interest, in a prospective observational study, patients with tumour-related neuropathic pain components (93 out of 302 patients) were more likely to achieve a pain response after radiation therapy administration than those without such component [48]. In this scenario, multifraction radiation therapy may be preferred to a single fraction, as it leads to longer durability of pain control [31]. It is associated with a higher remineralization rate of irradiated vertebrae, in comparison to a single fraction [17].

**Figure 3:**
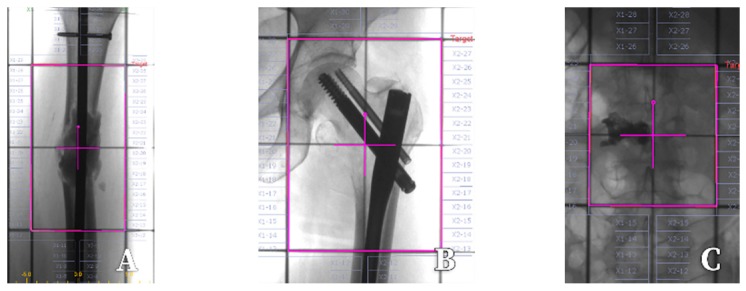
Simulation fields (pink rectangle) for irradiation of painful bone metastases with megavoltage photon external beams. (**A**,**B**)—metastases to the femur after surgical stabilization, (**C**)—metastasis to the vertebral body after percutaneous vertebroplasty.

In case of spine metastases, combining percutaneous vertebroplasty (decreasing pain through restoring mechanical stability of vertebrae) with radiation therapy (exhibiting antitumor effect) provides better pain relief than radiotherapy alone [49] (Figure 3).

In a scenario of spinal cord or cauda equina compression caused by a metastatic mass, direct decompressive surgery followed by radiation therapy seems to be superior to radiotherapy alone [50]. The usual dose is 20 Gy delivered in 5 days or 30 Gy given in 10 days [15]. In patients who are unfit for surgical management, radiation therapy is indicated to alleviate the pain and decrease neurologic complications [15], although the optimal dose and radiation regimen are not established yet. Taking into account medical emergency in spinal cord compression, a shorter radiation schedule is desirable. Results of a randomized controlled trial, performed in a group of 276 patients with life expectancies of fewer than 6 months, indicate that both regimens (8 Gy × 2 days and multifraction split regimen: 5 × 3 Gy and 5 Gy × 3) are equally efficient (pain relief in 56% vs. 59% patients, motor capacity in 68% vs. 71% patients), thus the short course should be recommended as the treatment of choice in the group of patients [51]. In patients with bone fractures, surgical management along with radiation therapy should be performed (Figure 2).

It should also be depicted that the histology of a metastatic tumour influences its radiosensitivity and the analgesic effect of radiation therapy. Arcangeli et al. [52] demonstrated that non-small cell lung cancer metastases appeared to be the least responsive among all primary tumours, with 46% complete pain relief in comparison to bone metastases from breast and prostate cancers (65%, *p* = 0.04 and 83%, *p*-0.002, respectively).

In summary, the decision on RT fractionation should be supported by the American Society for Radiation Oncology (ASTRO) Evidence-Based Guideline [53]. The panel states there exists pain equivalency following a single 8 Gy fraction, 20 Gy in 5 fractions, 24 Gy in 6 fractions, and 30 Gy in 10 fractions for patients with previously unirradiated painful bone metastases. Patients should be made aware that single fraction RT is associated with a higher rate of re-treatment to the same painful site than in fractionated treatment. A single fraction treatment may be particularly convenient and sensible for patients with limited life expectancy. Patients who experience persistent or recurrent pain more than one month following external beam radiation therapy (EBRT) for symptomatic peripheral bone metastases or in spine lesions should be considered for re-treatment while adhering to normal tissue dosing constraints described in the available literature [53].

#### 2.2.2. Hemibody Irradiation (HBI)

In patients with multiple painful bone metastases, hemibody irradiation using external beams may be a treatment option. The upper, middle or, lower half of the skeleton may be irradiated. Both photon beams and electron beams may be used [54,55]. Delivering 6–8 Gy in single fraction results in pain response in 70% of patients after 24–48 h [54]. The intensity of pain is reduced significantly, from 8 to 1, according to the visual-analogue scale, and there is a decrease in morphine consumption [54,55]. It is a very convenient treatment type for advanced cancer patients since it involves a short hospital stay and acceptable side effects [54].

#### 2.2.3. Stereotactic Surgery/Stereotactic Body Radiation Therapy

As was mentioned earlier, since conventionally delivered radiation therapy leads to pain relief in approximately 80% of patients, but complete pain alleviation is only experienced by 25–30% of them, another concept of radiation therapy has emerged. Furthermore, the oligometastatic state in clinical practice has been defined when 1–5 distant metastases are found in a cancer patient [56]. Advances in radiation therapy planning software, custom patients immobilization, and sophisticated radiation delivery equipment utilizing multiple conformal beams or arc therapy with intensity modulation and image guidance, with each treatment allowing for accuracy within millimetres, facilitate obtaining a very precisely covered radiation target with extremely high biological doses of radiation delivered in 1 to few fractions [57] (Figure 2C). Single fraction radiation therapy is called “radiosurgery” whereas delivering multiple fractions—stereotactic body radiation therapy (SBRT). The latter is alternatively called stereotactic ablative radiotherapy since a huge biological dose of radiation is delivered to the treated target. Indications for SBRT include one to three vertebral metastases, less than 5 cm in diameter [52]. Symptomatic spinal cord compression may be a contraindication for the treatment, as the myelopathy after SBRT may increase [57]. Several doses per fraction are used, from single 15–24 Gy fractions to 18–36 Gy delivered in 1–5 fractions [57,58,59,60,61,62]. Of note, data concerning efficacy and safety of the treatment come from retrospective analyses [46,47,48,49,50,51]. Nevertheless, SRS/SBRT is widely used in most radiation therapy centres. About 40% of radiation oncologists in the US report that they use spine SBRT in their radiation departments and the single fraction is preferable due to greatest patient comfort and outpatient treatment convenience [63]. Unfortunately, many studies assessing the efficacy of spine SBRT report excellent local control (LC approximately 90%), while they do not provide information on pain control [57].

Most studies report that pain control continues for 1-year after spine SBRT in as many as 84–90% of patients [58,59,60,61,62,64] and about 50% of patients experience complete pain relief at 6 months [61,65].

To date, the optimal dose and fractionation schedule of SRS/SBRT is unclear. Chang et al. [58] reported pain control in 89.2% of patients suffering from different types of tumours. Ryu et al. [59], in a phase II dose escalation trial, demonstrated that a dose above 14 Gy in a single fraction was associated with increased pain control (one-year actuarial pain control was 84%, whereas 46% of patients experienced complete pain relief, 18.9%—partial relief, and 16.2% reported stable pain intensity). Currently, a RTOG 0631 phase 2/3 randomized study compares pain relief between 8 Gy in a single fraction given with conventional radiotherapy versus 16 Gy in a single fraction delivered with stereotactic techniques [66]. Sprave et al. [60], in a single-institution randomized explorative trial on a group of 55 patients suffering from painful spinal metastases, documented that after single-fraction SRS (24 Gy), patients achieved quicker and improved pain responses, in comparison to 3DCRT (30 Gy in 10 fractions). McGee et al. [67], in a retrospective analysis of 96 patients undergoing SRS to the spine metastases from primary tumours of radioresistant histology (hepatocellular cancer, cholangiocarcinoma, renal cell carcinoma, melanoma, or leiomyosarcoma), found high rates of pain relief (93%), but pointed out that hepatocellular carcinoma was associated with an inferior response to radiation therapy. The concept of single fraction SRS has some limitations. The most important one is the risk of postradiation myelopathy since vertebral masses are in close proximity to the spinal cord, which is characterized by a limited dose of tolerance [62,68]. In cases of radiosurgical treatment, the epidural tumour component is the most frequent site of treatment failure, since the spine and epidural tumour mass was spared from a high dose of radiation [58,59,60,62,66,67,68]. Thus, a distance of more than 3 mm from the tumour mass and spinal cord is desired [66].

Fractionated SBRT to spine metastases (27 Gy in 3 fractions) resulted in pain relief in 52% of renal cancer patients after one year [61]. An 84% improvement in symptomatic patients was also observed in a study performed by Gibbs et al. [62]. In a retrospective, international, and multicentre study based on 387 spinal metastases treated with SBRT (median total dose—24 Gy in 3 fractions) it was demonstrated that worse outcomes of radiation therapy were associated with an interval between primary tumour diagnosis and SBRT smaller than 30 months and the presence of histology of primary cancer, such as non-small cell lung cancer, renal cell cancer, and/or melanoma [69]. Prior to the treatment, patients were pain-free or reported pain of mild/moderate or severe intensity in 18.2%, 64.9%, and 16.9%, respectively. The patients remained pain-free at the time of the last clinical assessment (median follow-up of 11.5 months) in 76.8%, 56.3%, and 43.8%, respectively [64,69]. According to ASTRO guidelines, advanced radiation techniques, such as SBRT as the primary treatment for painful spine bone lesions, should be considered in the setting of a clinical trial or with data collected in a registry, given that insufficient data is available to routinely use this treatment [53].

#### 2.2.4. Re-Irradiation

Palliative radiation therapy of bone metastasis for recurrent pain after previous palliative radiotherapy may be administered. It depends, however, on the location, prior radiation dose, fractionation schedule, and the time between radiation treatments [70]. Results obtained by Chow et al. [36], in a randomized trial, demonstrated that retreatment with a single fraction for painful bone metastases produces an effect equal to a multifraction conventional radiation therapy. The second conventional radiation treatment should be given no earlier than after 1 month [36]. Response to re-irradiation with conventional radiation therapy, however, was modest, with overall response rates of 45–52% and complete pain relief experienced by only 11–14% of patients [36]. These indicate a need for more effective re-treatment, and SBRT offers such opportunity [71,72]. A systematic review of studies concerning SBRT in re-irradiation of spine masses proved safety and good results in terms of local control and pain relief, although data is of low-quality or limited [72]. Only some studies reported results concerning pain control [58,73,74,75,76,77]. (Table 3).

In the case of previously irradiated, but progressing spinal metastases, re-irradiation should be ordered with caution, since the spinal cord is a radiosensitive structure [78,79]. The risk of myelopathy after re-irradiation is presumably low if the following conditions are met: The cumulative dose is less 135.5 Gy_2_, the interval between treatments is more than 6 months, and no course of radiation therapy exceeds the dose of 98 Gy_2_ [70,80] Sahgal et al. [64] found that an interval between conventional palliative radiation therapy and SBRT re-irradiation longer than 5 months, when maximum point dose to the thecal sac is limited to nBED 20–25 Gy (2/2), appears to be safe. Furthermore, the cumulative point max to the thecal sac should not exceed 70 Gy (2/2) and the SBRT thecal sac point max dose should not be above 50% of the total cumulative dose [64]. According to ASTRO guidelines, advanced radiation techniques, such as SBRT re-treatment for recurrent pain in spine bone lesions, may be feasible, effective, and safe, but this approach should be limited to clinical trial participation or on a registry given limited data supporting routine use [53].

This review is concentrated on radiation influence on pain control in cancer patients, but one has to realise that the final decision on qualification of patients to the SRS/SBRT should be taken at a multidisciplinary tumour board, assisted by the oncologic, neurologic, mechanical, and systemic framework defined by Laufer et al. [81], which allows for personalization of therapy.

## 3. Brachytherapy for the Treatment of Painful Bone Metastases

Brachytherapy consists in the application of radioactive sources inside the patient’s body (directly to the tumour burden or to the postoperative tumour bed) temporarily or permanently, which is meant to damage cancer cells’ DNA and destroy their ability to divide and grow. It allows for the use of a higher total dose of radiation to treat a smaller area in less time than the conventional external beam radiation therapy. Brachytherapy is a rarely used treatment option for bone metastases. Recently, a systematic review was published, which summarized the role of the treatment modality in painful spinal metastases [82]. Seven studies (which analysed treatment efficacy on pain control) reported a decrease of pain intensity after brachytherapy [83,84,85,86,87,88,89] (Table 4).

### Pain Flare Syndrome

Pain relief contributes to improved quality of life of cancer patients, which is currently one of the most important goals of the treatment. Radiation therapy is widely used for decreasing pain in the population, however, in some patients “pain flare” can occur after this treatment. It is observed in 2–40% of patients [90,91] and is defined as a temporary increase of bone pain at the treated site during radiation therapy or early after its cessation [92]. Although the precise mechanism of this phenomenon is not recognized yet, biochemical mediators of inflammation, which are released upon the radiation therapy or transient oedema compressing nerves at the site of treatment, are suggested to contribute to this toxicity [93]. Steroids decrease pain flare intensity [28]. Furthermore, anti-inflammatory medications may prevent or reduce the risk of toxicity.

## 4. Radioactive Isotopes for the Treatment of Painful Bone Metastases

**Figure 4:**
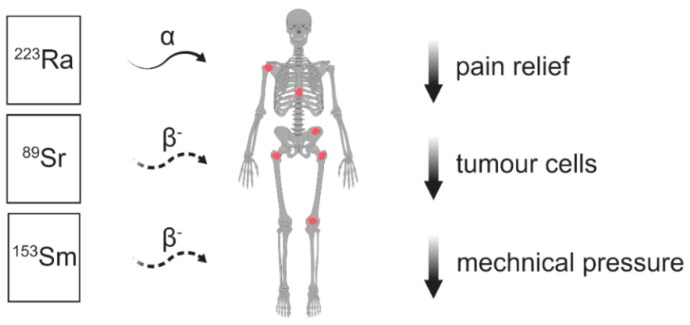
Usage of radioactive isotopes for multiple bone metastases. Abbreviations: ^223^Ra—Radium-223, ^89^Sr—Strontium-89, ^153^Sm—Samarium-153, α—alpha particle, β^−^—beta-minus particle.

In cancer patients suffering from multiple bone metastases, bone-seeking radiopharmaceuticals have proven to be an effective alternative [94,95,96,97,98] (Figure 4, Table 5). Strontium-89 chloride, Samarium-153-ethylenediamine tetramethylene phosphonic acid (EDTMP), Rhenium-186-hydroxyethylidine diphosphonic acid (HEDP), and Radium-233 dichloride have been approved for the treatment of bone pain due to osteoblastic or mixed bone metastases, mainly from prostate and breast cancers (most common indications) and other tumours presenting with painful osteoblastic lesions, confirmed by whole-body bone scintigraphy performed within at least 8 weeks before therapy [98,99,100,101]. These agents mainly accumulate in osteosclerotic and osteoblastic bone metastases, whereas they are not suitable for treating osteolytic and osteoclastic bone metastases. Furthermore, in metastatic bones vulnerable to fracture, local therapy such as surgery or radiation therapy should be performed prior to radionuclide therapy [102].

**Figure 5:**
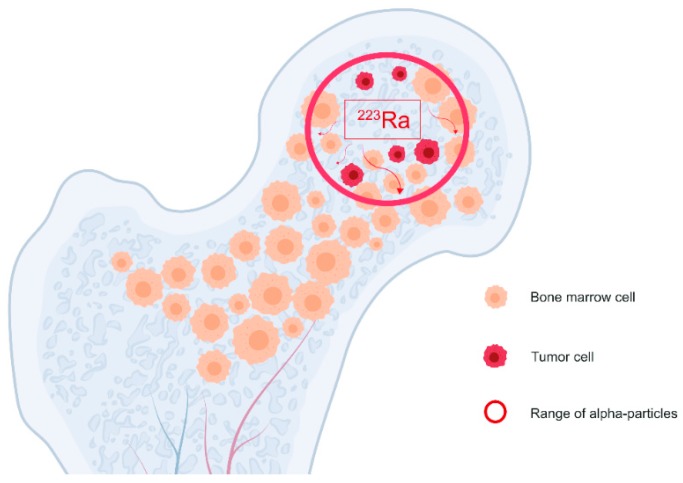
Local bone analgesic activity of alpha radiation delivered with Radium-223-dichloride.

The mechanism of pain palliation resulting from beta particles or alpha particles emitted by radionuclides is not clear yet. Beta or alpha particles kill tumour cells; therefore, pain relief occurs because of mechanical pressure reduction (Figure 5). Pain control, however, frequently occurs before the tumour mass shrinks. Lymphocytes, which are radiation sensitive cells, secrete a variety of cytokines causing pain. The death of lymphocytes cells at the tumour site may also contribute to pain relief [94].

### 4.1. Several Radionuclides Are Used in Clinical Practice, and Many of Them Are under Investigation

#### 4.1.1. Strontium-89 Chloride

There is solid data on the efficacy of Strontium-89 Chloride for bone pain relief in patients with prostate and breast cancers, with a pain relief rate of 63–88% [94,103,104,105,106,107,108,109,110]. Symptomatic improvement usually occurred within 6 weeks after administration, with a mean duration of the pain-free period of about 6 months [111], with no dose-response relationship [112]. Retreatment for responders is possible at time intervals of not less than 12 weeks [112,113].

#### 4.1.2. Samarium-153-EDTMP

Reduction of bone pain occurs in 62–78% of patients with bone metastases within 1 week of Samarium-153-EDTMP administration, with a definite dose-response relationship [109,114,115,116,117,118,119,120,121], with a mean duration of approximately 3–8 months. In several phase II/III clinical trials this radionuclide has shown significant efficacy for bone pain alteration in patients with various types of cancer, including lung, prostate, and breast cancer as well as osteosarcoma [122,123,124]. The minimum interval for retreatment should be 8 weeks [112,113].

#### 4.1.3. Radium-223-Dichloride

A phase III randomized double-blind placebo-controlled trial, ALSYMPCA, investigated the effectiveness of Radium-223-dichloride in 921 patients with metastatic castration-resistant prostate cancer with symptomatic bone metastases, previous use of analgesics or radiotherapy to bones, and no visceral metastasis. There was a significant improvement in median overall survival in the Radium-223-dichloride group vs. the placebo group (14.9 vs. 11.3 months respectively) and median time to the first symptomatic skeletal event (15.6 vs. 9.8 months, respectively) [125,126,127]. Median time to initial opioid use was significantly longer in the Ra-223 group, with risk reduction of 38%, compared to placebo. Less Ra-223 patients (36%) than placebo patients (50%) required opioids for pain relief. The QOL pain score showed reduced pain for Ra-223 patients relative to placebo patients at week 16 [128].

#### 4.1.4. Rhenium-186-HEDP

Clinical studies assessing Rhenium-186-HEDP were mainly performed on patients with prostate cancer and breast cancer [129,130,131,132,133]. General response rates for pain palliation ranged between 38% and 82% [95,99,134,135,136,137,138,139]. A pain response occurred 1–3 weeks after administration, with a durability of 5–12 months. No definite relationship between dose and pain response was observed [138]. Retreatment for responders is possible at time intervals of not less 6–8 weeks [140,141].

#### 4.1.5. Rhenium-188-HEDP

In a study by Palmedo et al. [142], with Rhenium-188-HEDP pain relief occurred in 64% of prostate cancer patients with bone metastases. Mean duration of the effect was 7.5 weeks [142]. In a study by Liepe et al. [143], pain relief was achieved in 76% of patients with hormone-refractory prostate carcinoma treated with the radionuclide. Therefore, in patients with progressive hormone-resistant prostate carcinoma and bone pain, repeated Rhenium-188-HEDP administration revealed the pain response rate of 92% vs. 60% and durability of response of 5.66 months, compared to 2.55 months for the single treatment group, respectively [144].

### 4.2. Side Effects of Radionuclide Therapy

The above-mentioned agents may produce some side effects such as gastrointestinal ulceration, enhanced bleeding, neutropenia, and disturbed renal function [94]. In about 10% of cases, regardless of the agent used, there is a possibility of a flare (a painful response with an increase of pain insensitivity). Usually, within 72 h of administration, these symptoms (typically temporary, mild, and self-limiting) should be gone. When osseous metastases involve the cervical spine, a low risk of post-therapy spinal cord compression exists and prophylactic corticosteroids should be given [97,98,99].

## 5. Radiation Therapy for Painful Primary/Regional/Metastatic Solid Tumours Other Than Bone Tumours

Progressing soft tissue tumours may also produce mild to severe pain. Primary or metastatic brain tumours, particularly those presenting with a larger mass effect or surrounding oedema, are frequently associated with pain. Surgery and radiotherapy are under consideration in the group of patients, depending on different factors (performance status of the patient, presence of uncontrolled extracranial disease, expected survival, etc.). Brain radiation therapy, among others—whole brain radiation therapy—may help reduce tumour mass and volume of oedema, thus leading to pain relief. Lung cancer patients may suffer from severe pain resulting from the invasion of brachial plexus by the direct apical tumour. Meta-analysis of 14 randomized clinical trials evaluating palliative radiation therapy for lung cancer patients revealed satisfactory symptomatic relief, among others—pain reduction [150]. Various fractionation schedules were used in the trials as follows: 10 Gy in 1 fraction, 17 Gy in 2 fractions, and 20 Gy in 5 fractions, as well as long courses, such as 30–45 Gy in 10–15 fractions [150]. Locally advanced gastrointestinal cancer patients may also suffer from the pain of different intensity. Administration of IMRT for a gastric tumour in patients with satisfactory performance status leads to a decrease of symptoms, among others—pain—in more than 70% of patients [151]. Pain may be experienced by advanced rectal cancer patients. Palliative radiation therapy to the pelvis, both using conventional fractionation (45 Gy in 25 fractions) and hypofractionated radiotherapy (30 Gy in 6 fractions) produces satisfactory pain relief in approximately 70% of patients [152,153]. As many as 40% of pancreatic cancer patients are diagnosed with locoregionally advanced disease or progress during the course of the disease [154]. The infiltration of nerves in the area surrounding the pancreas is observed in 43–72% of patients, which causes severe pain [155]. Delivering median 28 Gy (25–33 Gy) in 5 fractions using SBRT resulted in abdominal pain relief in 78% of advanced pancreatic cancer patients [156].

## 6. Pain Assessment after Radiation Therapy

There is no uniform pain assessment in cancer patients receiving radiation therapy. Some authors report pain control as subjective physician/patient reports [75,76,102,157]. Validated Brief Pain Inventory (BPI) was also used [158]. The International Bone Metastases Consensus Working Party proposed pain response categories in palliative radiation therapy [159]. They defined four response categories:

1. Complete response: A pain score of 0 at the treated site and no concomitant increase in analgesic intake, which means stable or reduced analgesics in daily oral morphine equivalent (OMED).
2. Partial response: Pain decrease of 2 or more at the treated site on a scale of 0 to 10 without analgesic increase, or analgesic dose decrease of 25% or more from the baseline without an increase in pain intensity.
3. Pain progression: Increase in pain score of 2 or more above the baseline at the treated site with stable OMED, or an increase of 25% or more OMED in comparison to the baseline with the pain score stable or 1 point above the baseline.
4. Intermediate response: Any response that is not captured by those defined above [159].

## 7. Future Directions

New and high-quality prospective data is awaited that will answer several questions and allow for definite statements regarding different combinations of radiation therapy (IMRT, SBRT, brachytherapy, and radionuclide therapy) with surgery (kyphoplasty or vertebroplasty for spine metastases or intramedullary fixation or endoprosthetic reconstruction for long bone metastases) and/or novel molecularly targeted agents/immunotherapy. Prospective phase III randomized studies will define the optimal use (in terms of efficacy and toxicity) of SBRT for treatment of newly diagnosed or recurrent painful spinal metastases. The STEREO-OS trial, which assesses the effect of standard systemic treatment in oligometastatic (3–5 sites) prostate, breast, and/or lung cancer patients in combination with SBRT for painful bone metastases [160], may serve as an example. Biological image-guided SBRT for painful bone metastases, with non-homogenous dose escalation based on FDG-PET(^18^F-Fluorodeoxyglucose—positron emission tomography) results, is another interesting concept [161]. Delivering high biological dose in the tumour region using IMRT with integrated boost for painful spinal bone metastases is the subject of the IRON-2 trial (Intensity-modulated Radiotherapy With Integrated-boost in Patients With Spinal Bone Metastases) [162]. Furthermore, the VERTICAL study is ongoing, assessing the analgesic effect of SBRT comparing to standard low dose EBRT [163]. Prospective cohorts of patients with painful bone metastases (the PRESENT study—Prospective Evaluation of Interventional Studies on Bone Metastases) or exclusively with long bone metastases (the OPTIMAL study) are formed to better guide personalized treatment in terms of improved quality of life and analgesic effect of radiation therapy, surgery, or combined treatment modalities [164,165].

A novel concept is a radiosurgical hypophysectomy for intractable bone metastases pain. Australian researchers will assess whether delivery of a single high dose (150 Gy) of radiation therapy to a small area of the pituitary gland and pituitary stalk in a highly precise manner may be helpful in reducing intractable pain from bone metastases [166].

The Palliative Radiotherapy and Inflammation Study (PRAIS) aims to find predictive factors associated with inflammation for palliative RT for cancer-induced pain response [167]. Furthermore, the latest findings over genetic biomarkers of different aspects of palliative RT for painful bone metastases are very interesting. Namely, Furfari et al. [168,169,170] identified genes’ profiles for changes in quality of life and pain relief, pain flare, and dexamethasone response following RT.

Development of higher quality data will further help find the best combinations of EBRT with bisphosphonates, radiopharmaceuticals, and novel biological agents reducing formation/activity of osteoclasts and bone resorption, like monoclonal antibody directed against RANKL—Receptor Activator for Nuclear Factor κ B Ligand (e.g., denosumab or novel agents, like JMT103) [171,172] or reducing cancer-induced bone osteolysis c-src inhibitors (dasatinib, bosutinib) [173].

## 8. Conclusions

Radiation therapy plays an important role in pain relief in cancer patients. As the radiation oncology field evolved, a number of challenges appeared. Important issues exist which have to be resolved, such as the following: Inconsistent endpoints of trials, difficulty in measuring the response, reluctance to practice evidence-based medicine (e.g., the choice of optimal regimen, re-treatment fractionation), differences in physicians’ and patients’ perspectives, as well as incorporating systemic treatment in combination with radiation therapy (bisphosphonates, nanotechnology, etc.).

Evidence-based treatment guidelines should be established and followed. Collaboration in multidisciplinary tumour boards provides the best, personalized, holistic care, which leads to, among others, pain reduction and an improved quality of patients’ life.

## Figures and Tables

**Table 1:** Factors influencing the radiation technique and fractionation schemes in the treatment of painful bone metastases in clinical practice.

Patient-Related Factors	Tumour-Related Factors	Logistic Issues
Performance status Patient’s mobility Clinical circumstances painful uncomplicated bone metastases pathologic fractures spinal cord compression re-irradiation Compliance to treatment Life expectancy Socioeconomic status Patient’s preferences Pain intensity	Histologic type of primary tumour Time elapsed from primary diagnosis to bone metastases Multiplicity of metastases Time of developing pain or neurologic deficits before RT ^1^	Treatment duration Significance of family members’ assistance Hospital location Distance from patient’s home to radiation therapy department Cost of therapy Reimbursement issues Organization of radiation departments Availability of multidisciplinary tumour board

^1^ Radiation therapy.

**Table 2:** Clinical studies demonstrating the analgesic effect of radiation therapy on painful bone metastases depending on dose and fractionation regimen.

Trial	Number of Patients	Fractionation	Complete or Partial Pain Response	Complete Pain Response
Price et al., 1986 [29]	288	1 × 8 Gy 10 × 3 Gy	73% 64%	45% 28%
Cole et al., 1989 [30]	29	1 × 8 Gy 6 × 4 Gy	88% 85%	NR ^2^ NR ^2^
Gaze et al., 1997 [26]	280	1 × 10 Gy 5 × 4.5 Gy	84% 89%	39% 48%
Nielsen et al., 1998 [25]	241	1 × 8 Gy 5 × 4 Gy	44% 46%	15% 17%
Steenland et al., 1999 [24]	1171	1 × 8 Gy 6 × 4 Gy	72% 69%	37% 33%
Koswig et al., 1999 [17]	107	1 × 8 Gy 10 × 3 Gy	79% 82%	31% 33%
BPTWP ^1^ 1999 [22]	272	1 × 8 Gy 5 × 4 Gy	72% 68%	52% 51%
Roos 2005 [31]	275	1 × 8 Gy 10 × 3 Gy	61% 53%	15% 18%
Hartsell et al., 2005 [32]	998	1 × 8 Gy 10 × 3 Gy	65% 66%	15% 18%
Foro Arnalot et al., 2008 [23]	160	1 × 8 Gy 10 × 3 Gy	75% 86%	15% 13%
Nongkynrih et al., 2018 [27]	60	1 × 8 Gy 5 × 4 Gy 10 × 3 Gy	80% 75% 85%	20% 20% 20%

^1^ BPTWP—Bone Pain Trial Working Party, ^2^ NR—not reported.

**Table 3:** Pain response after re-irradiation of painful spine metastases [58,73,74,75,76,77].

Study	Initial RT Dose (Median)	Re-Irradiation Modality	Re-Treatment Dose	Pain Response
Choi et al., 2010 [73]	40 Gy (24.2–50.4)	CyberKnife	Median marginal dose 20 Gy/2fr (range 18/1–25/5) 30 Gy/5fr	65% improvement in pain
Garg et al., 2011 [74]	30 Gy (30–45)	IG-IMRT	27 Gy/3fr 20 Gy/5fr	Improvement in pain at 6 months
Damast et al., 2011 [75]	30 Gy (8–66)	IG-IMRT	20 Gy/5fr 30 Gy/5fr	77% improvement in pain
Mahadevan et al., 2011 [76]	30 Gy (8–46)	CyberKnife	25–30 Gy/5fr 24 Gy/3fr	79% improvement in pain
Chang et al., 2012 [58]	39Gy Gy_2_^1^	CyberKnife	20.6 Gy/1fr (18.2–23.7)	80.8% pain control rate at 1 year
Hashmi et al., 2016 [77]	30 Gy/10fr	IG-IMRT	16.6 Gy/fr 24 Gy/3fr	74.3% improvement in pain

^1^ Gy is a way to normalize radiation doses that may have been given in a different dose/fractionation schedule. It converts all doses to the equivalent dose in 2 Gy per fraction. Abbreviations: fr—fraction, RT—radiation therapy, IG-IMRT—image-guided intensity modulated radiation therapy.

**Table 4:** Influence of brachytherapy on pain control in painful spine tumours [83,84,85,86,87,88,89]. ^125^I-iodine, 153Sm-samarium, SM—spinal metastases, VAS-visual-analogue scale of pain assessment.

Study	Brachytherapy Intervention	Pain Control VAS (Mean +/− Standard Deviation)	
		Pretreatment	Posttreatment
Cardoso et al., 2009 [83]	Percutaneous curettage of SM cement augmentation, and bone cement injection with ^153^Sm	8.5 +/− 2	2.6 +/− 3.1
Yang et al., 2013 [84]	Cement augmentation and percutaneous ^125^I seed implantation	8.73 +/− 0.31	1.32 +/− 0.37
Cao et al., 2014 [85]	Percutaneous ^125^I seed implantation	4.48 +/− 2.03	1.18 +/− 1.38
Huang et al., 2014 [89]	Cement augmentation and percutaneous ^125^I seed implantation	7.12 +/− 1.48	2.26 +/− 1.07
Li et al., 2014 [86]	Cement augmentation and percutaneous ^125^I seed implantation	7.7 +/− 1.3 (SN) 8.0 +/− 1.2 (MN)	2.6 +/− 1.0 2.4 +/− 1.1
Wang et al., 2015 [87]	Cement augmentation and percutaneous ^125^I seed implantation	6.37 +/− 1.67	1.32 +/− 0.75
Qian et al., 2016 [88]	Pedicle fixation of affected vertebra and implantation of ^125^I seeds via needles	7.43 +/− 0.98	4.29 +/− 0.98

Abbreviations: ^125^I—iodine, ^153^Sm—samarium, SM—spinal metastases, VAS-visual-analogue scale of pain assessment.

**Table 5:** Radioactive isotopes used for the treatment of painful bone metastases.

Radioactive Isotope	Trial	Study Type	Number of Patients	Cancer	Analgesic Effect	Duration of Analgesic Effect
Radium-223-Chloride	Nilsson et al., 2005 [145]	phase I	25 pts	breast, prostate	50% pts	1 WP 52%, 4 WP 60%, 8 WP 56%
	Bruland et al., 2006 [146]	phase I	6 pts	prostate	repeated administration was well tolerated	NS
	Nilsson et al., 2007 [147]	phase II randomized	64 pts	prostate	10 versus 16 (placebo) reported bone pain after injection	NS
	Coleman et al., 2014 [148]	phase IIa	23 pts	breast	BPI pain severity index at week 17 was 0.6	NS
	Parker et al., 2011 *ALSYMPCA* [127]	phase III randomized	921 pts	castration-resistant prostate	NS	NS
	Prelaj at al., 2019 [149]	retrospective	32 pts	prostate	71%	NS
Rhenium-186-HEDP	Maxon et al., 1991 [136]	double-blind	20 pts	prostate	80% pts	NS
	Maxon et al., 1992 [137]	prospective	43 pts	breast, prostate	77% pts initial injection 50% second in injection	7 weeks
	Han et al., 1999 [138]	prospective	30 pts	breast	58% pts	NS
	Han et al., 2002 *PLACORHEN* [139]	double-blind randomized	111 pts	prostate	0–96% (mean 27%)	NS
Rhenium-188-HEDP	Palmedo et al., 2000 [142]	prospective	22 pts	prostate	64%	7.5 weeks
	Liepe et al., 2000 [143]	prospective	15 pts	prostate	76%	NS
Samarium-153-EDTMP	Dolezal et al., 2000 [114]	prospective	33 pts	prostate, breast, other	70%	NS
	Wang et al., 2003 [115]	Comparative randomized	9 pts	prostate, breast, other	78%	3.5 +/− 2.3 months
	Sapienza et al., 2004 [116]	retrospective	73 pts	prostate, breast	76%	NS
Samarium-153-EDTMP	Etchebehere et al., 2004 [117]	retrospective	58 pts	prostate, breast, other	78%	5.75–6 months
	Sartor et al., 2004 [118]	phase III randomized	152 pts	prostate	64%	NS
	Tripathi et al., 2006 [119]	prospective	86 pts	prostate, breast, other	73%	2–8 months
	Ripamonti et al., 2007 [120]	prospective	13 pts	prostate, breast	61,5%	NS
	Liepe et al., 2007 [109]	prospective	15 pts	prostate, breast	73%	10 +/− 1 weeks
	Dolezal et al., 2007 [121]	prospective	32 pts	prostate	72%	3 months
Strontium-89 Dichloride	Sciuto. et al., 2001 [104]	randomized	51 pts	breast	84%	2–14 months
	Turner et al., 2001 [105]	prospective	93 pts	prostate	63%	NS
	Dafermou et al., 2001 [106]	multicentre observational	527 pts	prostate	59.8%	5.0 +/− 3.5 months
	Ashayeri et al., 2002 [107]	prospective	27 pts	prostate, breast	81%	up to 1 year
	Baczyk et al., 2003 [108]	prospective	70 pts	prostate	88%	3–12 months
	Fettich et al., 2003 [103]	prospective	93 pts	bone mts	75%	NS
	Liepe et al., 2007 [109]	prospective	15 pts	prostate, breast	72%	9 +/− 2 weeks
	Ma et al., 2008 [94]	prospective	116 pts	prostate	83.6%	3–12 months
	Zenda et al., 2014 [110]	prospective	54 pts	26 pts prostate/breast 28 pts other malignancies (lung, head and neck, colorectal, other)	69.2% 73.1%	2–6 months

Abbreviations: PTS—patients, NS—not stated, WP—week point, BPI—Brief Pain Inventory, MTS—metastases.
